# Setup and Validation of a Reliable Docking Protocol for the Development of Neuroprotective Agents by Targeting the Sigma-1 Receptor (S1R)

**DOI:** 10.3390/ijms21207708

**Published:** 2020-10-18

**Authors:** Giacomo Rossino, Marta Rui, Luca Pozzetti, Dirk Schepmann, Bernhard Wünsch, Daniele Zampieri, Giorgia Pellavio, Umberto Laforenza, Silvia Rinaldi, Giorgio Colombo, Laura Morelli, Pasquale Linciano, Daniela Rossi, Simona Collina

**Affiliations:** 1 Department of Drug Sciences, Medicinal Chemistry and Pharmaceutical Technology Section, University of Pavia, V.le Taramelli 12, 27100 Pavia, Italy; giacomo.rossino01@universitadipavia.it (G.R.); marta.rui01@universitadipavia.it (M.R.); luca.pozzetti01@universitadipavia.it (L.P.); pasquale.linciano@unipv.it (P.L.); daniela.rossi@unipv.it (D.R.); 2 Institute of Pharmaceutical and Medicinal Chemistry, University of Münster, Corrensstraße 48, 48149 Münster, Germany; dirk.schepmann@uni-muenster.de (D.S.); wuensch@uni-muenster.de (B.W.); 3 Department of Chemical and Pharmaceutical Sciences, University of Trieste, 30126 Trieste, Italy; dzampieri@units.it; 4 Department of Molecular Medicine, Human Physiology Unit, University of Pavia, via Forlanini 6, 27100 Pavia, Italy; giorgia.pellavio01@universitadipavia.it (G.P.); lumberto@unipv.it (U.L.); 5 SCITEC-CNR, via Mario Bianco 9, 20131 Milano, Italy; Silvia.rinaldi24@gmail.com; 6 Dipartimento di Chimica, Università di Pavia, V.le Taramelli 12, 27100 Pavia, Italy; g.colombo@unipv.it; 7 Department of Medical Biotechnology and Translational Medicine, University of Milan, Via Saldini 50, 20133 Milan, Italy; Laura.Morelli@unimi.it

**Keywords:** Sigma-1 receptor, docking, molecular modelling, neuroprotection, neurodegeneration, acetylcholinesterase, aquaporins, oxidative stress

## Abstract

Sigma-1 receptor (S1R) is a promising molecular target for the development of novel effective therapies against neurodegenerative diseases. To speed up the discovery of new S1R modulators, herein we report the development of a reliable in silico protocol suitable to predict the affinity of small molecules against S1R. The docking method was validated by comparing the computational calculated *K_i_* values of a test set of new aryl-aminoalkyl-ketone with experimental determined binding affinity. The druggability profile of the new compounds, with particular reference to the ability to cross the blood–brain barrier (BBB) was further predicted in silico. Moreover, the selectivity over Sigma-2 receptor (S2R) and *N*-methyl-d-aspartate (NMDA) receptor, another protein involved in neurodegeneration, was evaluated. 1-([1,1’-biphenyl]-4-yl)-4-(piperidin-1-yl)butan-1-one (**12**) performed as the best compound and was further investigated for acetylcholinesterase (AchE) inhibitor activity and determination of antioxidant activity mediated by aquaporins (AQPs). With a good affinity against both S1R and NMDA receptor, good selectivity over S2R and favorable BBB penetration potential together with its AChE inhibitory activity and its ability to exert antioxidant effects through modulation of AQPs, **12** represents a viable candidate for further development as a neuroprotective agent.

## 1. Introduction

Neurodegenerative diseases (which include Alzheimer’s diseases, Parkinson’s disease, amyotrophic lateral sclerosis and multiple sclerosis, among others) are unanimously considered one of the most important and difficult therapeutic challenges of our time [1,2]. The lack of effective therapies is due to the complex multifactorial nature of these pathologies, which involve the alteration of several molecular pathways, eventually leading to loss and impairment of neurons [1,2,3].

**Figure 1:**
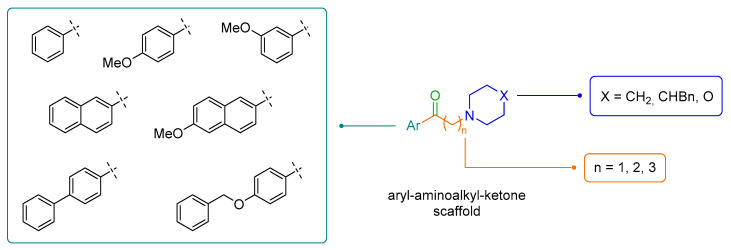
General scaffold of the published aryl-aminoalkyl-ketone series [15] and elements of structural differentiation suitable for the development of new analogs.

For several years Sigma-1 receptor (S1R) has attracted the attention of medicinal chemists as potential target for treating neurodegenerative diseases. The endogenous ligand of S1R has not been unanimously identified yet and along the years it has been discovered that S1R can be activated by some endogenous compounds, like neuropeptides, neurosteroids, N,N-dimethyltryptamine, or by physiological signals like oxidative or endoplasmic reticulum (ER) stress [4,5,6,7]. S1R is a transmembrane chaperone protein, present in the ER, nucleus and plasma membranes but particularly enriched in mitochondria-associated ER membranes (MAMs) [8,9,10]. Modulation of S1R may have a relevant potential from a therapeutic standpoint. In fact, S1R agonists possess neuroprotective and neuroplastic activities, since they modulate several molecular cascades, including calcium homeostasis regulation, glutamate excitotoxicity inhibition and oxidative stress [9,11,12,13]. The latter is a typical condition in debilitating pathologies where S1R is involved, like neurodegenerative diseases. In fact, neurodegenerative diseases, such as Alzheimer’s and Parkinson’s diseases as well as amyotrophic lateral sclerosis share common features, and these pathological cellular mechanisms develop in different ways, but all ultimately lead to apoptosis, necrosis, synapse and cell loss [1,3,14]. In our effort to discover new chemical entities against neurodegeneration, we recently disclosed two compounds as dual S1R modulators/acetylcholinesterase (AChE) inhibitors, characterized by the aryl-aminoalkyl-ketone structure reported in Figure 1 [15]. These compounds belong to a series of Multi Target Directed Ligands (MTDL) designed by combining the pharmacophoric elements of compound RC-33, the well-known AChE inhibitor Donepezil, and the antioxidant natural product Curcumin [15]. In particular, RC-33 is an in-house developed S1R agonist showing a nanomolar affinity against S1R and Sigma-2 receptor (S2R) (*K_i_*S1R = 1.8 ± 0.1 nM; *K_i_*S2R = 45 ± 16 nM) , a good selectivity over S2R, μ-, and κ-opioid receptors, a high metabolic stability in several biological matrices and a suitable pharmacokinetic profile and Central Nervous System (CNS) distribution for in vivo administration. In addition, RC-33 promotes the neurite differentiation and elongation in rat dorsal root ganglia (DRG) experimental model [15].

The principal aim of this work was to further investigate the potential of this scaffold, integrating computational and synthetic approaches to generate new molecules with optimized S1R binding affinities. The first step entailed developing a reliable docking protocol able to recapitulate the affinity of a series of known analogues for S1R and shed light on the most relevant pharmacophoric determinants for the interaction. Afterwards, we designed and synthesized a small library of new aryl-aminoalkyl-ketone (Figure 1) and evaluated the S1R binding affinity of the new molecules. From the correlation obtained upon comparing computational and experimental data, the efficacy of the proposed model was proved.

To identify compounds with potential for development into anti-neurodegenerative agents, the druggability, with particular reference to the ability to cross the blood–brain barrier (BBB), and the selectivity over S2R and *N*-methyl-d-aspartate receptor (NMDAR), another well-known molecular target involved in neurodegeneration, was investigated [16,17,18]. Lastly, we assayed the potential in restoring the damage induced by oxidative stress of the compound with the best profile. In detail, we evaluated the ability of our best compound to restore the aquaporin (AQP)-mediated water permeability in heat-stressed cells. AQPs are integral membrane proteins able to facilitate the diffusion of water and H_2_O_2_ from the producing cells across the plasma membranes to the extracellular fluid [19,20]. Since a crucial pathological event of neurodegenerative diseases is the oxidative stress, the possibility to chemically regulate the pore gating of AQPs, may provide a new direction to the development of new therapeutic treatments for degenerative diseases and in ageing [21,22].

## 2. Results

### 2.1. Setup and Validation of a Docking Model to Predict Affinity toward S1R

**Figure 2:**
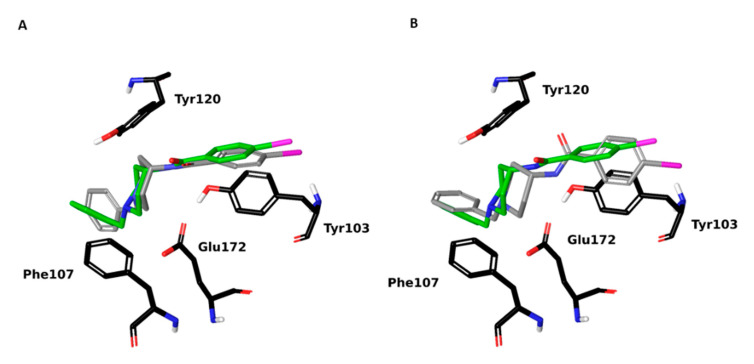
Difference of 4-IBP poses in ligand docking (**A**) and induced fit docking (**B**) from the crystal pose (shown in green color).

To develop a model able to correlate the structure of ligands to their ability to bind S1R, we docked ligands to the crystal structure (PDB ID: 5HK2) of the receptor published by Schmidt et al. [23]. As a first step towards a reliable protocol, we tested the combination of parameters that could best reproduce (with high accuracy) the X-ray structure of the complex between S1R and *N*-(1-benzylpiperidin-4-yl)-4-iodobenzamide (4-IBP). In this framework, the ligand was removed and subsequently redocked with the rigid docking procedure described in Material and Methods. After running the calculations, we evaluated the root mean square deviation (RMSD) of the positions of the heavy atoms of the ligand between the most favorable calculated pose and the crystallographic one. Importantly, the RMSD was as low as 0.9 Å, demonstrating that the docking protocol was able to reproduce the binding mode of crystallographic pose (see Figure 2A). Subsequently, the redocking procedure was re-examined using an induced fit docking (IFD) approach (see Material and Methods). This type of docking allows the binding pocket residues to adapt to the presence of the ligand, mimicking the protein-ligand cross-talk that expectedly takes place upon binding events. In this case, RMSD between the docked and the crystalized ligand increased to 1.6 Å due to small rearrangements of atoms and functional groups (the orientation of ketone group). However, it is worth pointing out that the overall binding mode is conserved, as shown by the superimposition of the two poses (see Figure 2B).

**Figure 3:**
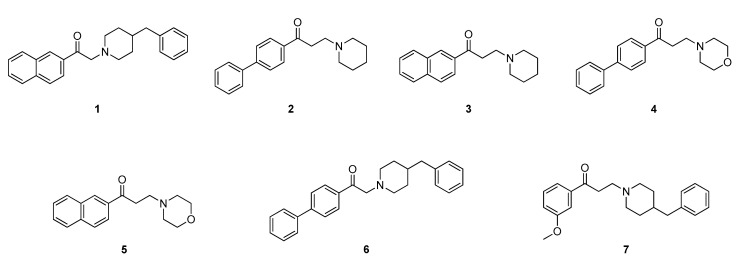
Chemical structures of the previously developed S1R ligands, which have been assessed in the present work for docking calculation.

Once established and validated by reproducing the binding properties of the experimental crystal structures, the IFD docking protocol was applied to representative compounds (**1**–**7**) previously synthetized and evaluated by our group (Figure 3). In particular, we selected compounds that exhibited different binding profiles to cover a wide range of *K_i_* values. Besides hit compounds emerged from our previous study (**1** and **2**) [15], we docked compounds **3**–**5** endowed with binding affinities lower than 10 nM, and compounds **6**, **7** which exhibited *K_i_* > 300 nM.

Overall, we considered 11 ligands because for compounds which had a p*K_a_* next to the physiologic one (**1**, **4**–**6**) we considered both states (dissociated and not). At the end of the runs, we compared results of Gscore based ligand efficiency (LE), a scoring function which describes the binding energy considering all chemical properties of atoms involved in the interaction [24], with *K_i_* values, under the hypothesis that the two parameters are correlated. LE normalizes the calculated binding energy as a function of the number of heavy atoms that make up the ligand. In this context, the Glide Gscore LE represents a proxy for affinity (see Table 1). For ligands that exist in different states in physiological conditions, we calculated the average of values.

**Figure 4:**
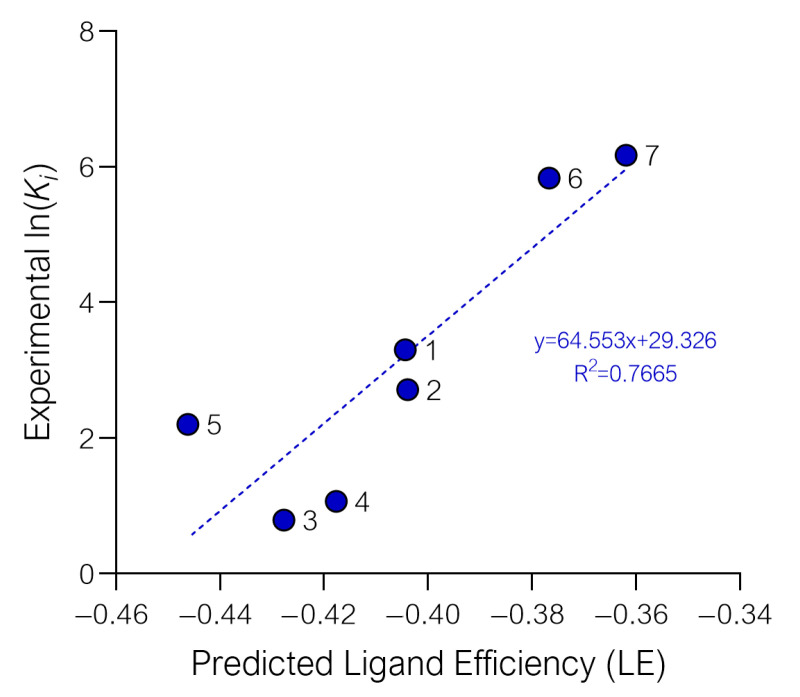
Correlation between experimentally determined ln(*K_i_*) and predicted ligand efficiency.

Importantly, the use of LE as the affinity parameter returned a good correlation with experimental affinity values (R^2^ = 0.77), as reported in Figure 4.

The data showed that compounds **1**–**5** were correctly located in a region that corresponds to a good ligand efficiency. Conversely, LE for **6**–**7** correctly predicted their worse performances (LE > −0.3800). This model correctly captures the difference between the weak affinity of compounds **6**–**7** (which are correctly placed in the region of the graph corresponding to bad performance) and the rest of the series, clustering compounds with *K_i_* in the range of tens nM (namely the two hit compounds **1**, **2**) differently from those with *K_i_* ≤ 9 nM. These results suggest that this approach can be proficiently used as a first screening of compound affinities for S1R. To corroborate this hypothesis, we designed a small new series of analogues with the purpose to calculate their predicted affinity and then to synthesize and evaluate their experimental *K_i_* through binding assays.

### 2.2. Design of New Ligands

**Figure 5:**
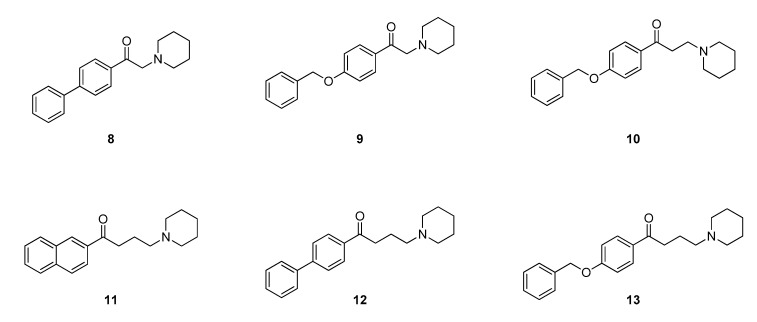
Chemical structures of the new aryl-aminoalkyl-ketone derivatives.

Among the two molecules previously emerged as the most promising ligands, compound **2** was chosen as the starting point to prepare a new library of ketones [15]. This choice is strictly related to a putative easier way to perform the future in vitro assays. The piperidine moiety confers a much higher solubility in the aqueous medium, thus improving the pharmacokinetic properties and the drug-likeness of the molecules. In the previous work, the piperidine ring was present only in the series of ketones with two carbon atoms in the linker (*n* = 2). For this reason, we decided to expand the library with compounds characterized also by a single carbon atom (*n* = 1) or by three carbon atoms (*n* = 3) linker. The aromatic moieties explored include naphth-2-yl, 4-biphenyl and 4-(benzyloxy)phenyl. In particular, this last group maintains a similar hydrophobicity as the other two moieties, but at the same time it possesses some new features able to give interesting information. Firstly, the presence of an oxygen atom, which can act as a H-bond donor, gives us insights about the actual hydrophobicity of this side of the binding pocket. Furthermore, we reasoned that we could further explore the binding pocket by elongating the molecule with the addition of another aromatic ring together with an sp^3^ carbon atom as a more flexible linker. The designed library consists of six different ketones reported in Figure 5. This compound library still maintains the structural features needed to interact with S1Rs, namely the basic nitrogen and the hydrophobic moieties [23,25].

The four major concerns in pharmacokinetics are absorption, distribution, metabolism and excretion (ADME). These four criteria influence the pharmacological activity of bioactive compounds and therefore their developability. In silico pharmacokinetic properties of the designed compounds **8**–**13** were predicted using QikProp program to assess a number of significant properties such as blood–brain barrier (BBB) permeability, polar surface area, human oral absorption percentage and molecular weight [26,27]. The results are reported in Table 2. Considering the p*Ka* of compounds **8**–**13**, compound **8** was computed as free base at physiological pH, whereas compound **9** was considered both as dissociated and undissociated species. All the other entries are protonated species. All values were found to be within the range of most approved drugs, meaning that our designed compounds are characterized by a good developability and drug-likeness. In particular, the QPlogBB value indicates the predicted brain/blood partition coefficient. QPlogBB values of compounds **8**–**13** fall between −3.0 and 1.2, which is the range of 95% of known drugs. Overall, the designed compounds are suited for targeting CNS receptors. Moreover, no violations to Lipinski’s rule of five or to Jorgensen’s rule of three were found [28,29].

### 2.3. Synthesis

**Scheme 1:**
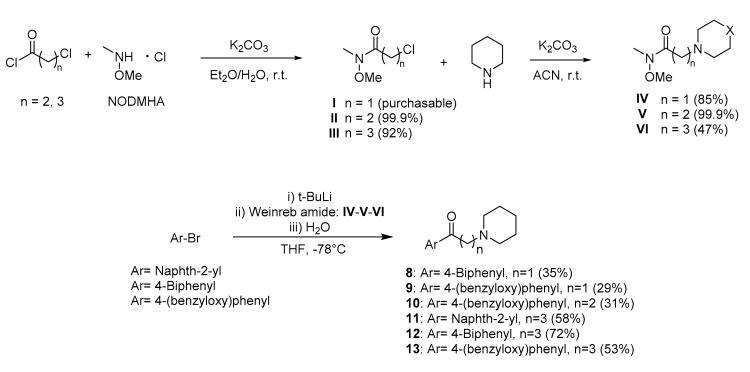
Synthetic route to achieve the target compounds **8**–**13**.

To obtain the designed compounds we adopted the same synthetic strategy reported in our previous work [15]. A divergent synthesis was exploited, as reported in Scheme 1. Key intermediates **IV**-**VI** were obtained through Weinreb amide formation and subsequent nucleophilic substitution. Finally, target compounds **8**–**13** were obtained via Weinreb ketone synthesis, using different aryl bromides.

All the designed compounds were obtained in suitable amount and purity for further biological investigation.

### 2.4. S1R Binding Profile 

Compounds **8**–**13** were docked on S1R using the same protocol and their ligand efficiencies were calculated. This allowed us to predict their *K_i_* values exploiting the equation of the correlation line reported in Figure 4. The obtained results, reported in Table 3, indicate that the new compounds are expected to exhibit an excellent binding profile, with affinity as low as sub-nanomolar. Even the less performing compound **13** still displays a predicted *K_i_* < 300 nM.

**Figure 6:**
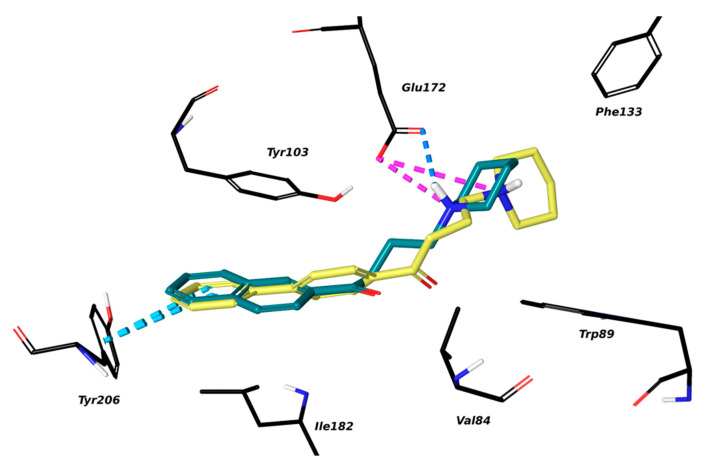
Binding mode of **11** (in turquoise sticks) and **12** (in yellow sticks). The main amino acids of the protein involved in the binding are represented as black wires. Dashed lines indicate the π stackings (in cerulean), the electrostatic interactions (in pink), and H-bonds (in blue). Atoms are colored-coded (oxygen in red, nitrogen in blue).

In particular, we compared the specific poses of best performing ligands, namely **11** and **12**. We noticed that **11** had better affinity because the protonated piperidine established an additional H-bond with Glu172 (see Figure 6). The formation of this additional interaction may be reconnected to the presence of naphthalene, a less cumbersome substituent than biphenyl. Naphthalene, indeed, allows a better adaptation of the molecule and, therefore, it induces the ligand to populate a more favorable orientation for the interaction.

**Figure 7:**
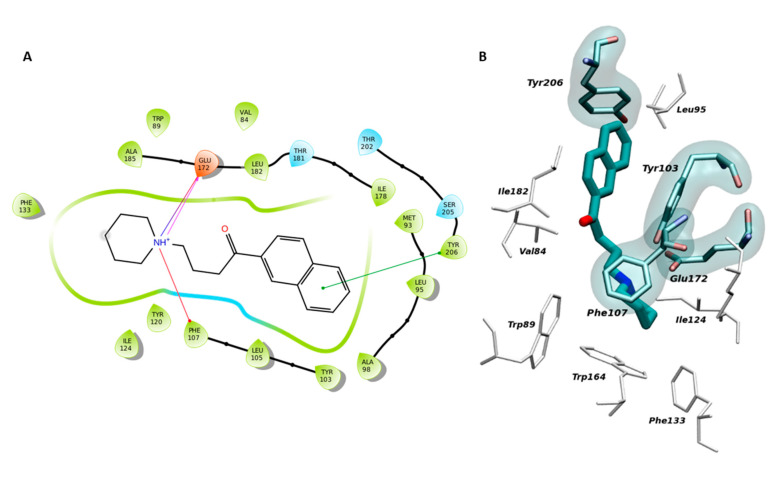
Binding mode of **11** in 2D space (**A**) and 3D space (**B**). The residues of the protein involved in hydrophobic interactions are reported in white sticks.

Overall, analyses of our models indicated that most of the ligands with protonated piperidine established an electrostatic interaction with Glu172 and a cation-π interaction with Phe107. This amino ring is further stabilized by weak interactions with another aromatic residue, Trp89. Moreover, the aromatic substituent establishes principally stacking interactions with Tyr103 and Tyr206. The pocket is purely hydrophobic (see Figure 7) and, therefore, the carbon skeleton establishes numerous additional hydrophobic interactions with the residues that line the pocket. All of these interactions contribute to stabilize the ligand pose.

Based on the docking correlation analysis and the inspection of the poses reported above, we hypothesize that the aromatic moiety, alkyl linker and the protonated basic substituent are fundamental pharmacophoric elements to ensure affinity for the target. In this framework, the presence of a longer alkyl linker (*n* = 2/*n* = 3) on the one hand favors interactions with pocket’s hydrophobic residues, while making the compound more flexible on the other hand. It is tempting to speculate that such flexibility would allow the amino substituent of the ligand to dynamically adapt to the binding pocket, which is in turn expected to undergo conformational adaptation to the presence of the ligand. Facilitating the exploration of a larger number of interactions, this could favor the efficient selection of a more stable pose. Moreover, the carbonyl group is not involved in any particular interaction with the binding pocket residues. This can actually be an advantage in the pursuit of multi-target ligands: The ketone moiety could be exploited for interaction with other molecular targets involved in neurodegeneration—such as NMDA receptor—without affecting binding to S1R.

To verify reliability of the developed docking protocol, we performed experimental binding assays toward S1R. Binding site affinities of compounds **8**–**13** were measured through competition experiments, using radioligands. The assay for S1R was performed using homogenized guinea pig cerebral cortex membranes, in the presence of [^3^H]-(+)-pentazocine, as a potent and selective S1R radioligand. Nonspecific binding values were determined using non-radiolabeled (+)-pentazocine and haloperidol in excess (see experimental section). The results are presented in Table 3.

**Figure 8:**
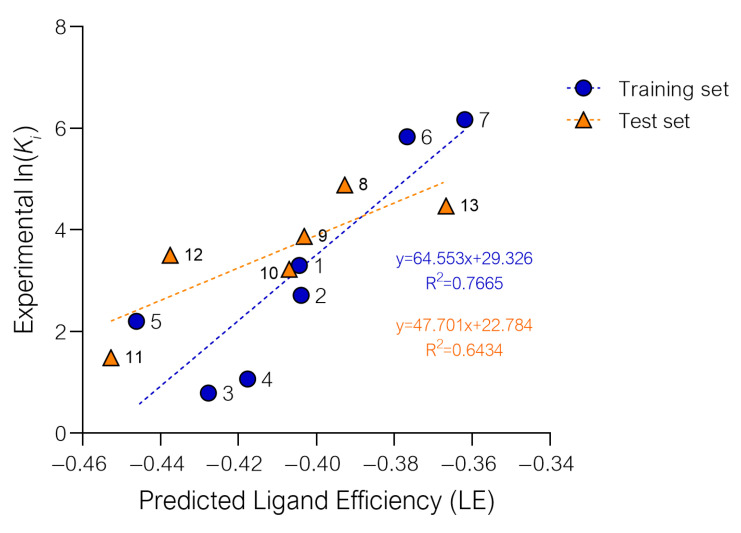
Correlation between calculated LE and experimental ln(*K_i_*). Blue color indicates the line, the equation and the R^2^ value for the correlation between LE and ln(*K_i_*) for the first data set compounds **1**–**7** employed for the model construction. With orange color are indicated the line, the equation and the R^2^ value for the correlation between LE and ln(*K_i_*) for the wider data set, including all compounds **1**–**13**.

The experimental affinities were compared with the predicted ones as reported in the graph of Figure 8. Blue dots represent already published compounds that were used to build the model, and the blue line is the correlation between LE and ln(*Ki*) for such compounds. The orange dots correspond to the new analogues, and the orange line indicates the correlation between predicted and experimental affinity once all compounds **1**–**13** are taken into account. Interestingly, the correlation coefficient indicates a good predictivity of the model (R^2^ = 0.64). It is worth noting that the orange correlation line is obtained from a wider and more comprehensive data set.

Overall, the model was able to place new compounds correctly into the proper region of ligand efficiency, separating best performing molecules from those displaying weaker receptor affinity. Moreover, the dataset employed covers a significant range of binding affinities, from low nanomolar to hundreds nanomolar, suitable for ligands that can be further developed and/or optimized.

### 2.5. In Depth Pharmacological Profile

All compounds have also been tested for their affinity towards S2R and NMDAR. Homogenized rat liver membranes were adopted to evaluate the S2R binding values, employing [^3^H]-DTG—a non-selective S2R radioligand—and, non-tritiated (+)-pentazocine to mask the S1R. Compounds with high affinity were tested twice. For compounds with low affinity, only one measure was performed. Affinity towards GluN2 subunit of NMDA was determined through competitive binding assays on membrane extracts of L cells (tk-), stably transfected with a vector containing the genetic information of GluN1a and GluN2B subunits. [^3^H]-Ifenprodil was employed as a selective and potent GluN2 inhibitor radioligand. Compounds with high affinity were tested three times. For compounds with low NMDA affinity, only one measure was performed. A negligible affinity vs. S2R and NMDAR (*K_i_* S2R > 200 nM, *K_i_* NMDAR > 500 nM) was observed with the only exception of compound **11** (*K_i_* S2R = 50 nM) and compound **12** (*K_i_* NMDAR = 200 nM). Therefore, compound **12**, the only characterized by a good affinity for both S1R and NMDAR and no affinity for S2R was then selected for undergoing further in vitro investigation, by evaluating the AchE and antioxidant properties.

To test the potential to inhibit AChE, a spectrophotometric procedure was adopted, based on the well-known Ellman’s method [30]. Firstly, the target compound was tested at a concentration of 50 μM and a 72% of inhibition was observed. Accordingly, the IC_50_ value was determined, which resulted in 0.68 ± 0.09 μM.

**Figure 9:**
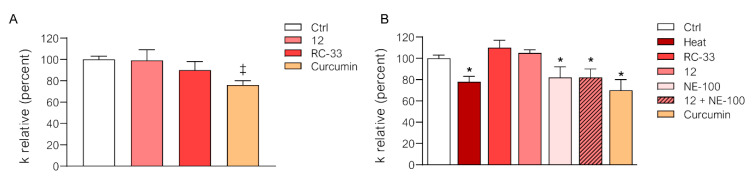
Effect of the compounds **12**, RC-33 and curcumin on the water permeability of HeLa cells in non-stressed condition (**A**) and in heat-stress condition (**B**). (**A**) Hela cells were incubated at 21 °C for 3 h with the compounds at the final concentration of 20 μM. Vehicle cells are incubated without compounds (white bars). Bars represent the osmotic water permeability of HeLa cells expressed as percent of k relative. (**B**) HeLa cells were exposed to a 150 mOsm osmotic gradient in three different conditions: (1) Untreated cells (Controls, Ctr); (2) cells treated at 42 °C for 3 h (heat-stressed, Heat); (3) heat-stressed cells pre-treated with the compound **12**, RC-33, NE-100, NE-100 + 12 combination, and curcumin at 20 μM final concentration. Values are means ± SEM of 4–15 single shots (time course curves) for each of 4–18 different experiments. * *p* < 0.05 vs. Ctr, **12**, RC-33; ‡ *p* < 0.05 vs. vehicle, **12**, RC-33 (ANOVA, followed by Newman–Keuls’s Q test).

Prompted by these encouraging results, we decided to draw the antioxidant profile of compound **12**, since it is well-known that oxidative stress is connected with insurgence and exacerbation of neurodegenerative diseases [3,18,31,32]. In particular, the AQP-mediated antioxidant properties of RC-33 (i.e., 1-[3-(1,1′-biphen)-4-yl]butylpiperidine, our in-house developed selective S1R agonist) and **12** were evaluated in HeLa cells, following the procedures reported in our most recent studies [22]. Briefly, osmotic water permeability was measured by a stopped-flow light scattering method and expressed as percentage of k relative. Compounds RC-33 and **12** were tested at 20 µM whereas curcumin was used as control of the goodness of the cellular assay, since its capability to affect the AQPs permeability was assessed in our previous work [22]. The compounds were initially assessed on non-stressed HeLa cells to evaluate the capability of the compounds to affect the AQPs permeability in absence of an oxidant stimuli. As reported in Figure 9A, compound **12** did not affect the water permeability and showed, as expected, a profile comparable to the parent compounds RC-33. Conversely, curcumin directly affect AQPs resulting in a significative permeability reduction (75% of k relative). RC-33, **12** and curcumin were further assessed under oxidative stress conditions induced by heating the cell cultures. As reported in Figure 9B, both RC-33 and **12** were able to prevent the water permeability reduction (values compared to the control), as instead significatively observed in cells offended by heating (80% of k relative). Notably, the curcumin was not able to protect the AQPs’ permeability properties in heat-treated cells. Thus, to probe the entanglement between S1R, AQP permeability and antioxidant protection, compound **12** was assessed against stressed HeLa cells in presence of NE-100, a well-known and fully characterized S1R antagonist. NE-100 was able to significatively quench the antioxidant effect of the S1R ligand **12**, with k relative values comparable to NE-100 tested as single agent (% of k relative of 78% and 80% for compounds NE-100 and the combination NE-100 + **12**, respectively). Thus, these results suggest that compound **12** acts as S1R agonist, exerting an AQP-mediated antioxidant effects by the engagement of S1R.

## 3. Discussion

In this work, we developed a docking protocol able to predict the S1R affinity of new aryl-aminoalkyl-ketone. Firstly, a reliable docking protocol was set up and validated. The co-crystallized ligand 4-IBP has been removed from the crystal structure and redocked using both ligand docking and induced fit docking (Figure 2). Results showed that both protocols were able to reproduce the binding mode of the crystallized ligand. A series of our previously synthesized SR ligands with aminoalkyl-ketone scaffold (**1**–**7**) were used as training set. These compounds covered a wide range of binding affinities (1 < *K_i_* < 500 nM). Regarding compounds with a p*K_a_* close to the physiologic one (i.e., ligands **1**, **4**–**6**), protonated species and free bases have been considered. Gscore based Ligand Efficiency (LE) resulted in the most valuable parameter to predict ligand’s affinity. As a result, a linear correlation between LE values and the experimental *K_i_* was observed with a R^2^ = 0.77 (Figure 4). Importantly, compounds in the same range of affinity were correctly clustered together. On the wave of the results obtained, the docking protocol was validated by designing and synthesizing a test set of six structurally related compounds, namely **8**–**13**. Moreover, the in silico ADME parameters were calculated. All designed compounds **8**–**13** resulted endowed with good BBB permeability, oral bioavailability. In addition, no violations to Lipinski’s rule of five and Jorgensen’s rule of three supported their druggability. Compounds **8**–**13** have then been docked on S1R using the abovementioned protocol and their LE scores calculated. Based on the equation reported in Figure 4, the *K_i_* values were predicted. In details, sub-nanomolar *K_i_* was predicted for compound **11**, while *K_i_* values in the tens of nanomolar were predicted for other compounds, with the only exception of compound **13**. The S1R binding assay showed *K_i_* values in accordance with the predicted ones. The satisfying correlation between LE and the experimental *K_i_* values testified the goodness of our docking protocol to predict the binding affinity of potential S1R ligands based on aminoketone scaffold. Overall, compounds **8**–**12** can be considered promising S1R ligands and were selected for further investigation.

The binding profile has been therefore extended by evaluating the affinity towards S2R and NMDAR. All compounds showed a negligible affinity vs. S2R and NMDAR with the only exception of compound **11** (*K_i_* S2R = 50 nM) and compound **12** (*K_i_* NMDAR = 200 nM). Importantly, NMDAR is strictly related to synaptic plasticity and synapse formation, which are key mechanisms underpinning learning and memory. Dysregulation of NMDAR activity leads to excitotoxicity and cell death, and is thus considered a potential mechanism of neurodegeneration [16,17,18]. Taking into account the relevant role of both S1R and NMDAR in neurodegenerative diseases, compound **12**, was then selected for undergoing further in vitro investigation, by evaluating the AchE and antioxidant properties. In fact, it is well known that dysfunction of neurotransmitter pathways (such as acetylcholine impairment) and oxidative stress are distinctive features of neurodegenerative conditions [18,31,33,34]. Furthermore, compounds exerting antioxidant effects are commonly regarded as attractive therapeutic tools for the treatment of neurodegenerative disorders. In fact, the brain is deficient in antioxidant defense systems and it requires a high level of oxygen, it is highly vulnerable to oxidative stress. AQPs are a family of water channels that play a role in scavenging reactive oxygen species, alleviating oxidative injury, and counteracting neuropathies [35,36]. These still poorly explored proteins have only recently been recognized as druggable molecular targets [37,38,39]. In our recent investigations, we have identified a number of molecules (both natural and synthetic) that are able to modulate AQPs and thus prevent cell damage due to oxidative stress [22]. Results obtained showed that compound **12** possesses anti-AChE activity (IC_50_ = 0.68 ± 0.09 μM) and it is able to prevent water permeability decrease in heat-stressed HeLa cells. In particular, combination studies with NE-100 (a S1R antagonist) suggested that the S1R agonist is able to exert an AQP-mediated antioxidant effects.

To sum up, in our ongoing quest for new chemical entities against neurodegeneration, we recently reported on aryl-aminoalkyl-ketone, as dual S1R modulators/AChE inhibitors endowed with antioxidant properties [15]. To speed up the identification of other promising neuroprotective agents, in the present work we developed a reliable computational protocol to predict affinity of novel compounds toward S1R. The proposed model is simple and efficient and can be proposed for designing new derivatives. Overall, the docking protocol herein proposed appears to capture the principal determinants for S1R binding, proving its immediate transferability to the design of new viable leads whose activity is experimentally supported. Our combined approach thus expands the chemical space of S1R ligands, in parallel generating new opportunities for testing in multitargeted strategies. Among the small series of novel compounds developed in the present work, we identified compound **12** as the most promising, being endowed with good affinity toward both S1 and NMDA receptors, good selectivity over S2R and favorable BBB penetration potential. Moreover, its AChE inhibitory activity and its ability to exert antioxidant effects through modulation of AQPs make **12** a viable candidate for further development as a neuroprotective agent and can be moved to in vivo studies.

## 4. Materials and Methods

### 4.1. Laboratory Materials and Equipment

Reagents and solvents for synthesis and deuterated solvents for NMR were purchased from Sigma Aldrich. Silica gel for flash chromatography (60 Å; 230–400 Mesh) was purchased from Sigma Aldrich. Solvents were evaporated at reduced pressure with the Heidolph Laborota 4000 Efficient equipment. Analytical thin layer chromatography (TLC) analyses were carried out on silica gel pre-coated glass-backed plates (TLC Silica Gel 60 F254; Merk) impregnated with a fluorescent indicator and visualized with the instrument MinUVIS DESAGA^®^ Sarstedt-GRUPPE by ultraviolet (UV) radiation from UV lamp (λ = 254 and 366 nm) or by stain reagents such as Ninidrine. NMR were measured at room temperature (15–25 °C) on a Bruker Avance 400 MHz and/or 500 MHz spectrometer, using tetramethylsilane (TMS) as internal standard and a BBI 5 mm probe. All raw FID files were processed with Top Spin program from Bruker and the spectra analyzed using the MestReNova 6.0.2 program from Mestrelab Research S.L. Chemical shifts are expressed in parts per million (ppm; δ scale). ^1^H-NMR spectroscopic data are reported as follow: chemical shift in ppm; multiplicity; coupling constants *J* (in Hz), and integration intensity.

The multiplicities are abbreviated as s (singlet), d (doublet), t (triplet), q (quartet), m (multiplet) and brs (broad signal). The chemical shift of all symmetric signals is reported as the center of the resonance range. ^13^C-NMR spectroscopic data are reported as follows: chemical shift in ppm.

### 4.2. General Experimental Details

All the following reactions were performed with dry glassware, previously flamed with Bunsen burner, fitted with rubber septum, under an atmosphere of nitrogen and with magnetic stirring. Liquid reagents, air-/moisture- sensitive and dry solvents were added using plastic syringes with metal needle, previously conditioned with nitrogen. Solid reagents were transferred opening the rubber septum under nitrogen flow or solubilizing them in appropriate dry solvents.

Low temperatures were reached with cooling agents, such as ice (0 °C), mixture of ice, methanol (MeOH) and sodium chloride (NaCl) (−18 °C), or mixture of solid carbon dioxide and acetone (−78 °C) placed in a Dewar suitable for the reaction flask.

Reactions progress and ending were monitored by TLC; in addition, the final products were analyzed with ^1^H and ^13^C Nuclear Magnetic Resonance (NMR). Purity of final compounds was assessed by UPLC-UV-ESI/MS. Analyses were carried out on a Acuity UPLC Waters LCQ FLEET system using an ESI source operating in positive ion mode, controlled by ACQUIDITY PDA and 4 MICRO (Waters). Analyses were run on a ACQUITY BEH Phenyl (ABP) (50 2.1 mm, 1.7 mm) or ACQUITY BEH Shield (ABS) (100 2.1 mm, 1.7 mm) columns, at room temperature, with gradient elution (solvent A: Water containing 0.1% of formic acid; solvent B: Methanol containing 0.1% of formic acid; gradient: 10% B in A to 100% B in 3 min, followed by isocratic elution 100% B for 1.5 min, return to the initial conditions in 0.2 min) at a flow rate of 0.5 mL min^−1^.

### 4.3. Synthetic Procedures

#### 4.3.1. General Procedure for the Preparation of Compounds **II**–**III**

To an aqueous solution of potassium carbonate (K_2_CO_3_, 2.0 equiv.) was added diethyl ether (Et_2_O) and *N*,*O*-dimethylhydroxyamine (NODMHA) hydrochloride (1.5 equiv.). The resulting mixture was cooled at 0 °C and then the corresponding acyl chloride (1.0 equiv.) was added dropwise. The reaction was let to reach room temperature and then it was stirred overnight. The reaction mixture was extracted with Et_2_O (2 × 10 mL) and washed with water (10 mL) and brine (20 mL). The organic phase was dried (anhydrous sodium sulphate Na_2_SO_4_), filtered and, after removal of the solvent under reduced pressure, the pure compounds **II**–**III** were obtained.

*3-Chloro-N-methoxy-N-methylpropanamide* (**II**)

By following the General Procedure, starting from 3-chloropropanoyl chloride (127 mg, 1.00 mmol, 1.0 equiv.), K_2_CO_3_ (276 mg, 2.0 mmol, 2.0 equiv.), *N*,*O*-dimethylhydroxyamine hydrochloride (146 mg, 1.5 mmol, 1.5 equiv.), H_2_O (3 mL) and Et_2_O (3 mL), the desired product was obtained in 92% (139 mg) as a pale yellow oil. **^1^H NMR** (500 MHz, CDCl_3_): δ 3.80 (t, *J* = 6.9 Hz, 2H, CH_2_CH_2_Cl), 3.70 (s, 3H, NOCH_3_), 3.19 (s, 3H, NCH_3_), 2.91 (t, *J* = 6.7 Hz, 2H, CH_2_CH_2_CO). **^13^C NMR** (125 MHz, CDCl_3_): δ 170.8, 61.4, 39.2, 35.0, 32.0.

*4-Chloro-N-methoxy-N-methylbutanamide* (**III**)

By following the General Procedure, starting from 4-chlorobutanoyl chloride (141 mg, 1.00 mmol, 1.0 equiv.), K_2_CO_3_ (276 mg, 2.0 mmol, 2.0 equiv.), *N*,*O*-dimethylhydroxyamine hydrochloride (146 mg, 1.5 mmol, 1.5 equiv.), H_2_O (3 mL) and Et_2_O (3 mL), the desired product was obtained in a quantitative amount (yield >99.9%) (166 mg) as a bright yellow oil. **^1^H NMR** (500 MHz, CDCl_3_): δ 3.70 (s, 3H, NOCH_3_), 3.63 (t, *J* = 6.4 Hz, 2H, CH_2_CH_2_Cl), 3.18 (s, 3H, NCH_3_), 2.62 (t, *J* = 6.7 Hz, 2H, CH_2_CH_2_CO), 2.11 (m, *J* = 6.9 Hz, 2H, CH_2_CH_2_CH_2_). **^13^C NMR** (125 MHz, CDCl_3_): δ 170.8, 61.4, 39.2, 35.0, 32.0.

#### 4.3.2. General Procedure for the Preparation of Compounds **IV–V–VI**

To a solution of the appropriate Weinreb amide **I**–**III** (1.0 equiv.) in acetonitrile (ACN), piperidine (1.0 equiv.) and K_2_CO_3_ (1.5 equiv.) were added. In case of compound **IV** the mixture was stirred for 36 h at room temperature; for compound **V** four days at room temperature were needed; lastly for compound **VI** the temperature was raised to 50 °C for 8 h, then it was let to reach room temperature and stirred for five days. After removal of the solvent under reduced pressure, the crude was extracted with dichloromethane (DCM) (3 × 5 mL) and washed with water (5 mL) and brine (10 mL). In the case of compound **IV**, this work-up was sufficient to obtain the pure compound. Conversely, an acid (pH = 3–4)/base (pH = 8–9) work-up was required for **V** and **VI**, the combined organic phases were dried over anhydrous Na_2_SO_4_, filtered and, evaporated under vacuum to get the desired compounds.

*N-methoxy-N-methyl-2-(piperidine-1-yl)-acetamide* (**IV**)

By following the General Procedure, starting from 2-chloro-N-methoxy-N-methylacetamide (**I**, commercially available) (138 mg, 1.00 mmol, 1.0 equiv.), K_2_CO_3_ (207 mg, 1.5 mmol, 1.5 equiv.), piperidine (175 mg, 176 mL 1.0 mmol, 1.0 equiv.) and ACN (10 mL), the desired product was obtained in 85% yield (235 mg) as a dark oil. **^1^H-NMR** (500 MHz, CDCl_3_) δ: 3.71 (s, 3H, NOCH_3_), 3.29 (s, 2H, CH_2_N), 3.19 (s, 3H, NCH_3_), 2.50 (m, 4H, CH_2_ piperidine), 1.71 (m, 4H, CH_2_ piperidine), 1.47 (m, 2H, CH_2_ piperidine).

*N-methoxy-N-methyl-3-(piperidine-1-yl)-propanamide* (**V**)

By following the General Procedure, starting from 3-chloro-N-methoxy-N-methylpropanamide (152 mg, 1.00 mmol, 1.0 equiv.), K_2_CO_3_ (207 mg, 1.5 mmol, 1.5 equiv.), piperidine (85 mg, 99 mL, 1.0 mmol, 1.0 equiv.) and ACN (7 mL), the desired product was obtained in 56% yield (112 mg) as a transparent oil. **^1^H NMR** (400 MHz, CDCl_3_): δ 3.76 (s, 3H, NOCH_3_), 3.24 (s, 3H, NCH_3_), 2.76 (s, 4H, CH_2_N, CH_2_CH_2_N), 2.52 (t, 4H, pip), 1.70–1.56 (m, 6H, pip). **^13^C NMR** (100 MHz, CDCl_3_): δ 175.2, 61.3, 54.5, 54.2, 29.7, 25.9, 24.2.

*N-methoxy-N-methyl-4-(piperidin-1-yl)-butanamide* (**VI**)

By following the General Procedure, starting from 4-chloro-N-methoxy-N-methylbutanamide (166 mg, 1.00 mmol, 1.0 equiv.), K_2_CO_3_ (207 mg, 1.5 mmol, 1.5 equiv.), piperidine (85 mg, 99 mL, 1.0 mmol, 1.0 equiv.) and ACN (7 mL), the desired product was obtained in 47% yield (101 mg) as a yellow oil. **^1^H-NMR** (400 MHz, CDCl_3_) δ: 3.70 (s, 3H, NOCH_3_), 3.16 (s, 3H, NCH_3_), 2.56 (t, *J* = 2.7 Hz, 2H, CH_2_N), 2.51 (m, 4H, CH_2_N piperidine), 2.19 (t, *J* = 7.4Hz, 2H, COCH_2_), 1.93 (m, 2H, COCH_2_CH_2_), 1.71 (m, 4H, CH_2_ piperidine), 1.46 (m, 2H, CH_2_ piperidine).

#### 4.3.3. General Procedure for the Preparation of Compounds **8–13**

Under nitrogen atmosphere, tert-butyllithium (2.5 equiv., 1.7 M in pentane) was added dropwise to a −78 °C cooled solution of the appropriate arylbromide (1.5 equiv.) in anhydrous tetrahydrofuran (THF). After 20 min, the solution of the corresponding Weinreb amide in anhydrous THF was added dropwise. The stirring was continued for 5 additional hours and then quenched with water. The reaction was extracted with Et_2_O (3 × 7 mL) and washed with water (5 mL) and brine (10 mL). The organic phase was dried over anhydrous Na_2_SO_4_, filtered and, after removal of the solvent under reduced pressure, the so-obtained crude mixture was subjected to chromatography (silica gel) to afford pure compound. Lastly, pure compound was converted into its corresponding hydrochloride, adding an ethereal solution of hydrogen chloride (HCl, 1.0 equiv., 1 M in Et_2_O).

*1-(2-([1,1’-biphenyl]-4-yl)-2-oxoethyl)piperidin-1-ium hydrochloride* (**8**)

By following the General Procedure, starting from 4-bromo-1,1′-biphenyl (1.5 equiv.), *N*-methoxy-*N*-methyl-2-(piperidin-1-yl)-acetamide (1.0 equiv.), t-BuLi (1.7 M, 2.5 equiv.) and THF, the desired product was obtained in 35% yield as a yellow solid after chromatography on silica gel (90:10 ethylacetate/methanol) and converted into the corresponding hydrochloride. 420 mg, pale yellow solid. **R_f_**: 0.27 (80:20 ethylacetate/methanol). **mp**: 236–238 °C. **^1^H NMR** (400 MHz, MeOD): δ 8.15 (d, *J* = 8.3 Hz, 2H, Ph-PhCO), 7.89 (d, *J* = 8.3 Hz, 2H, Ph-PhCO), 7.74 (d, *J* = 7.7 Hz, 2H, Biph), 7.53 (t, *J* = 7.5 Hz, 2H, Biph), 7.46 (t, *J* = 7.2 Hz, 1H, Biph), 4.99 (s, 2H, COCH_2_N), 3.65 (brs, 2H, pip), 3.16 (t, *J* = 13.1 Hz, 2H, pip), 2.08 – 1.87 (brs, 5H, pip), 1.69 – 1.55 (brs, 1H, pip). **^13^C-NMR** (100 MHz, MeOD) δ: 190.12, 147.42, 139.19, 132.31, 128.81, 128.67, 128.41, 127.17, 126.89, 61.19, 54.23, 22.57, 21.21. UHPLC-ESI-MS: ABP t_R_ = 3.98, 98% pure (λ = 294 nm), m/z = 281.1 [M + H]^+^.

*1-(2-(4-(benzyloxy)phenyl)-2-oxoethyl)piperidin-1-ium hydrochloride* (**9**)

By following the General Procedure, starting from 1-(benzyloxy)-4-bromobenzene (1.5 equiv.), *N*-methoxy-*N*-methyl-2-(piperidin-1-yl)-acetamide (1.0 equiv.), t-BuLi (1.7 M, 2.5 equiv.) and THF, the desired product was obtained in 29% yield as a yellow solid after chromatography on silica gel (80:20 ethylacetate/methanol) and converted into the corresponding hydrochloride. 400 mg, pale yellow solid. **R_f_**: 0.19 (80:20 ethylacetate/methanol). **mp**: 86–88 °C. **^1^H NMR** (400 MHz, MeOD): δ 8.03 (d, *J* = 8.8 Hz, 2H, OPhCO), 7.47 (d, *J* = 7.4 Hz, 2H, PhCH_2_), 7.41 (t, *J* = 7.3 Hz, 2H, PhCH_2_), 7.36 (d, *J* = 7.2 Hz, 1H, PhCH_2_), 7.19 (d, *J* = 8.8 Hz, 2H, OPhCO), 5.24 (s, 2H, PhCH_2_OPh), 4.87 (s, 2H, COCH_2_N), 3.61 (d, *J* = 12.1 Hz, 2H, pip), 3.11 (brs, 2H, pip), 1.94 (brs, 5H, pip), 1.60 (m, 1H, pip). **^13^C-NMR** (100 MHz, MeOD) δ: 193.52, 130.63, 128.58, 127.74, 115.10, 70.33, 59.24, 52.20, 23.04, 21.50. UHPLC-ESI-MS: ABP t_R_ = 4.12, 96% pure (λ = 285 nm), m/z = 311.2 [M + H]^+^.

*1-(3-(4-(benzyloxy)phenyl)-3-oxopropyl)piperidin-1-ium hydrochloride* (**10**)

By following the General Procedure, starting from 1-(benzyloxy)-4-bromobenzene (1.5 equiv.), *N*-methoxy-*N*-methyl-2-(piperidin-1-yl)-propanamide (1.0 equiv.), t-BuLi (1.7 M, 2.5 equiv.) and THF (5 mL), the desired product was obtained in 31% yield as a yellow solid after chromatography on silica gel (90:10 DCM/methanol) and converted into the corresponding hydrochloride. 560 mg, white solid. **^1^H NMR** (400 MHz, MeOD): δ 8.05 (d, *J* = 8.2 Hz, 2H, OPhCO), 7.47 (d, *J* = 6.3 Hz, 2H, PhCH_2_), 7.40 (t, *J* = 6.5 Hz, 2H, PhCH_2_), 7.35 (brs, 1H, PhCH_2_), 7.14 (d, *J* = 8.3 Hz, 2H, OPhCO), 5.22 (s, 2H, PhCH_2_O), 3.62 (brs, 2H, COCH_2_CH_2_), 3.55 (brs, 4H, pip), 3.04 (t, *J* = 12.5 Hz, 2H, COCH_2_CH_2_), 1.98 (brs, 2H, pip), 1.82 (brs, 3H, pip), 1.58 (brs, 1H, pip). **^13^C-NMR** (125 MHz, MeOD) δ: 194.91, 163.38, 136.55, 131.05, 129.00, 128.08, 127.49, 126.54, 113.90, 69.85, 53.31, 52.22, 32.29, 22.95. UHPLC-ESI-MS: ABP t_R_ = 4.03, 98% pure (λ = 263 nm), m/z = 324.4 [M + H]^+^.

*1-(4-(naphthalen-2-yl)-4-oxobutyl)piperidin-1-ium hydrochloride* (**11**)

By following the General Procedure, starting from 2-bromonaphthalene (1.5 equiv.), *N*-methoxy-*N*-methyl-4-(piperidin-1-yl)-butanamide (1.0 equiv.), t-BuLi (1.7 M, 2.5 equiv.) and THF, the desired product was obtained in 58% yield as a yellow solid after chromatography on silica gel (60:40 ethylacetate/methanol) and converted into the corresponding hydrochloride. 554 mg, white solid. **R_f_**: 0.17 (50:50 ethylacetate/methanol). **mp**: 213–215 °C. **^1^H NMR** (400 MHz, MeOD): δ 8.66 (s, 1H, Napht), 8.10 – 8.05 (m, 2H, Napht), 8.01 – 7.94 (m, 2H, Napht), 7.64 (m, 2H, Napht), 3.65 (d, *J* = 12.3 Hz, 2H, pip), 3.40 (t, *J* = 6.6 Hz, 2H, CH_2_CH_2_CH_2_), 3.28 – 3.20 (m, 2H, CH_2_CH_2_CH_2_), 3.01 (t, *J* = 12.4 Hz, 2H, pip), 2.29 – 2.17 (m, 2H, CH_2_CH_2_CH_2_), 2.01 (d, *J* = 14.9 Hz, 2H, pip), 1.86 (m, 3H, pip), 1.63 – 1.52 (m, 1H, pip). **^13^C-NMR** (125 MHz, CDCl_3_) δ: 199.1, 130.0, 129.7, 128.7, 128.5, 127.8, 126.1, 122.6, 56.7, 53.1, 35.6, 22.4, 22.1, 18.0. UHPLC-ESI-MS: ABP t_R_ = 3.77, 98% pure (λ = 248 nm), m/z = 282.0 [M + H]^+^.

*1-(4-([1,1’-biphenyl]-4-yl)-4-oxobutyl)piperidin-1-ium hydrochloride* (**12**)

By following the General Procedure, starting from 4-bromo-1,1′-biphenyl (1.5 equiv.), *N*-methoxy-*N*-methyl-4-(piperidin-1-yl)-butanamide (1.0 equiv.), t-BuLi (1.7 M, 2.5 equiv.) and THF, the desired product was obtained in 72% yield as a yellow solid after chromatography on silica gel (40:60 ethylacetate/methanol) and converted into the corresponding hydrochloride. 671 mg, white solid. **R_f_**: 0.23 (40:60 ethylacetate/methanol). **mp**: 250–251 °C. **^1^H NMR** (400 MHz, MeOD): δ 8.13 (d, *J* = 8.3 Hz, 2H, Ph-PhCO), 7.81 (d, *J* = 8.3 Hz, 2H, PhPhCO), 7.71 (d, *J* = 7.6 Hz, 2H, PhPhCO), 7.51 (t, *J* = 7.5 Hz, 2H, PhPhCO), 7.43 (t, *J* = 7.3 Hz, 1H, PhPhCO), 3.63 (brs, 2H, pip), 3.28 (t, *J* = 6.6 Hz, 2H, CH_2_CH_2_CH_2_), 3.25–3.18 (m, 2H, CH_2_CH_2_CH_2_), 3.00 (brs, 2H, pip), 2.25–2.13 (m, 2H, CH_2_CH_2_CH_2_), 1.92 (brs, 5H, pip), 1.59 (brs, 1H, pip).**^13^C-NMR** (125 MHz, CDCl_3_) δ: 198.8, 145.8, 139.7, 135.7, 130.0, 128.9, 128.6, 128.3, 127.3, 56.7, 53.2, 35.6, 22.5, 22.2, 18.0. UHPLC-ESI-MS: ABP t_R_ = 4.30, 97% pure (λ = 285 nm), m/z = 308.1 [M + H]^+^.

*1-(4-(4-(benzyloxy)phenyl)-4-oxobutyl)piperidin-1-ium hydrochloride* (**13**)

By following the General Procedure, starting from 1-(benzyloxy)-4-bromobenzene (1.5 equiv.), *N*-methoxy-*N*-methyl-4-(piperidin-1-yl)-butanamide (1.0 equiv.), t-BuLi (1.7 M, 2.5 equiv.) and THF, the desired product was obtained in 53% yield as a yellow solid after chromatography on silica gel (60:40 ethylacetate/methanol) and converted into the corresponding hydrochloride. 798 mg, white solid. **R_f_**: 0.18 (40:60 ethylacetate/methanol). **mp**: 188–190 °C. **^1^H NMR** (400 MHz, MeOD) δ: 8.02 (d, *J* = 8.8 Hz, 2H, OPhCO), 7.47 (d, *J* = 7.4 Hz, 2H, PhCH_2_), 7.40 (t, *J* = 7.3 Hz, 2H, PhCH_2_), 7.35 (d, *J* = 7.1 Hz, 1H, PhCH_2_), 7.12 (d, *J* = 8.8 Hz, 2H, OPhCO), 5.21 (s, 2H, PhCH_2_OPh), 3.60 (brs, 2H, pip), 3.18 (m, 4H, CH_2_CH_2_CH_2_), 2.98 (brs, 2H, pip), 2.21–2.08 (m, 2H, CH_2_CH_2_CH_2_), 2.04–1.72 (brs, 5H, pip), 1.57 (brs, 1H, pip).**^13^C-NMR** (125 MHz, CDCl_3_) δ: 198.1, 160.0, 138.1, 137.4, 130.1, 128.1, 127.7, 127.3, 114.4, 69.7, 56.3, 53.1, 31.2, 22.8, 21.5, 18.1. UHPLC-ESI-MS: ABP t_R_ = 4.32, 99% pure (λ = 273 nm), m/z = 338.0 [M + H]^+^.

### 4.4. Molecular Modelling on S1R

Docking analyses and calculations were carried out using the software Maestro, Schrodinger, LLC, New York, 2019, via the following protocol:

#### 4.4.1. Ligand Preparation

Each ligand is designed with 2D sketcher and then transferred into a 3D structure. Every ligand is prepared with LigPrep program of the MAESTRO suite from Schrodinger (release 2019-1; www.schodinger.com) considering a pH between 6 and 8 to mimic the physiological one. The force field for calculations was OPLS3 [40].

#### 4.4.2. Protein Preparation

As a receptor, we use the cocrystal-structure with PBD code 5HK2. Before starting calculations, the protein is prepared using the Protein Preparation Wizard program [41] of MAESTRO. Water molecules and all ligands (detergents etc.) except for 4-IBP are eliminated. The crystallographic structure is pre-processed adding hydrogens, create disulphide bonds where possible, assign bond orders, convert all selenomethionines into methionine and add missing/ill-resolved sidechains with Prime [42]. After completing the preparation of the structure, the protein is minimized using force field OPLS3 to remove bad contacts, take angle and dihedral values close to ideal, and remove excess strain.

#### 4.4.3. Grid Generation

This step is aimed to construct a grid that defines the shape and chemical properties of the targeted binding site. In particular, two fundamentals grids are generated: Bounding box and enclosing box. The bounding box circumscribes the site where ligand is free to move during the docking run. The enclosing box, on the other hand, is bigger and represents the delimited physical space where ligand atoms can move, to allow for potentially necessary rearrangements.

Before creating a grid, as an additional check of the capability of the program to correctly recognize binding sites, the SiteMap [43] program is used in a blind search starting from the protein structure devoid of ligand to detect the druggable site. SiteMap correctly returned to position of the actual binding site. Then, the energy grid is calculated using the Grid Generation tool of Maestro, defining the position of ligand 4-IBP as the centroid with a radius of 15 Å. Default Van der Waals scaling factor was used, with scaling factor of 1.0 and partial charge cut-off of 0.25.

#### 4.4.4. Ligand Docking

Ligand docking is carried out using the Glide program [44]. In this step, the ligand is located into the grid previously created with Grid Generation tool. Control docking experiments are carried out using the originally co-crystallized ligand 4-IBP in the corresponding crystal structure 5HK2 in order to validate and optimize docking parameter settings. The best docking pose obtained for 4-IBP correctly and accurately reproduces its crystal binding mode. Docking is performed employing Standard Precision (SP) mode, using OPLS3 force field and default Van der Waals scaling factor, with scaling factor of 0.80 and partial charge cut-off of 0.15. For each docking run 10 poses for ligand are saved.

#### 4.4.5. Induced-Fit Docking

The IFD protocol [45] includes docking of ligands with Glide Docking, Prime Refinement [46] and Glide Redocking of the complex and calculation of the binding score. In this case the centroid considered is the best pose (with the lowest GlideEmodel value) of the ligand which is obtained in Ligand Docking. Prime Refinement is performed on residues within 5 Å of the ligand pose. Glide Redocking is performed using standard precision (SP) scoring method of Glide, and the OPLS3 all-atom force field. Ten poses for ligand are saved for each run.

For each best pose, the Ligand Efficiency is calculated. This parameter normalizes the binding energy by considering the molecular size, which rescales the higher energy binding of ligands with high molecular weight, forming van der Waals and hydrophobic interaction [47].

### 4.5. General Protocol for Binding Assays

The test compound solutions were prepared by dissolving ≈10 µmol (usually 2–4 mg) of test compound in DMSO (unless otherwise specified), so that a 10 mM stock solution was obtained. To obtain the required test solutions for the assay, the DMSO stock solution was diluted with the respective assay buffer. The filtermats were pre-soaked in 0.5% aqueous polyethylenamine solution for 2 h at r.t. before use. All binding experiments were carried out in duplicate in 96-well multiplates. The concentrations given are the final concentrations in the assay. Generally, the assays were performed by addition of 50 µL of the respective assay buffer, 50 µL test compound solution at various concentrations (i.e., 10^−5^, 10^−6^, 10^−7^, 10^−8^, 10^−9^ and 10^−10^ M), 50 µL of corresponding radioligand solution, and 50 µL of the respective receptor preparation into each well of the multiplate (total volume 200 µL). The receptor preparation was always added last. During the incubation, the multiplates were shaken at a speed of 500–600 rpm at the specified temperature. Unless otherwise noted, the assays were terminated after 120 min by rapid filtration using the harvester. During the filtration each well was washed five times with 300 µL of water. Subsequently, the filtermats were dried at 95 °C. The solid scintillator was melted on the dried filtermats at 95 °C for 5 min. After solidifying the scintillator at r.t., the trapped radioactivity in the filtermats was measured with the scintillation analyzer. Each position on the filtermat corresponding to one well of the multiplate was measured for 5 min with the [^3^H]-counting protocol. The overall counting efficiency was 20%. The IC_50_ values were calculated with GraphPad Prism 3.0 (GraphPad Software, San Diego, CA, USA) by nonlinear regression analysis. The IC_50_ values were subsequently transformed into *K_i_* values using the equation of Cheng and Prusoff. The *K_i_* values are given as mean value ± SEM from three independent experiments.

#### 4.5.1. S1R Binding Assay

The assay was performed with the radioligand [^3^H](+)-pentazocine (22.0 Ci mmol^−1^; Perkin-Elmer). The thawed membrane preparation of guinea pig brain cortex (≈100 µg protein) was incubated with various concentrations of test compounds, 2 nM [^3^H](+)-pentazocine, and Tris buffer (50 mM, pH 7.4) at 37 °C. The nonspecific binding was determined with 10 mM unlabeled (+)-pentazocine. The *K_d_* value of (+)-pentazocine is 2.9 nM.

#### 4.5.2. S2R Binding Assay

The assay was performed using 150 µg of rat liver homogenate, which was incubated with various concentrations of test compound for 120 min at room temperature, along with 3 nM [^3^H]-DTG (Perkin-Elmer, specific activity 58.1 Ci mmol^−1^) in 50 mM Tris-HCl, pH 8.0, 0.5 mL final volume. (+)-pentazocine (100 nM) and haloperidol (10 µM) were used to mask S1R and to define nonspecific binding, respectively.

#### 4.5.3. GluN2 Binding Assay

The competitive binding assay was performed with the radioligand [^3^H]-ifenprodil (60 Ci mmol^−1^; BIOTREND, Cologne, Germany). The thawed cell membrane preparation from the transfected L(tk-) cells (about 20 µg protein) was incubated with various concentrations of test compounds, 5 nM [^3^H]-ifenprodil, and TRIS/EDTA buffer (5 mM TRIS/1 mM EDTA, pH 7.5) at 37 °C. The non-specific binding was determined with 10 mM unlabeled ifenprodil. The *K_d_* value of ifenprodil is 7.6 nM.

### 4.6. Inhibition of AChE

AChE inhibitory activity of compound **12** was determined by the modified Ellman’s method. Briefly, a stock solution of tested compound (5.0 mM) was prepared in DMSO and diluted using 0.1 M KH_2_PO_4_/K_2_HPO_4_ buffer (pH 8.0) to afford a final concentration range between 1 and 50 mM. Enzyme solutions were prepared by dissolving lyophilized powder in double-distilled water. The assay solution consisted of 845 mL of 0.1 M phosphate buffer KH_2_PO_4_/K_2_HPO_4_, 25 mL of AChE solution (0.22 U/mL, E.C. 3.1.1.7, from electric eel) and 10 mL of various concentrations of test compounds, which was allowed to stand for 5 min at 25 °C before 100 mL of 0.01 M DTNB were added. The reaction was started by addition of 20 mL of the 0.075 M substrate solution (acetylthiocholine iodide) and exactly 2 min after substrate addition, the absorption was measured at 25 °C at 412 nm. In enzyme-free assay systems the non-enzymatic hydrolysis of acetylthiocholine iodide was measured, and the results were employed as blank. In control experiments, inhibitor-free assay systems were utilized to measure the full activity. A positive control of Donepezil was used to afford a final concentration range from 10 nM to 50 µM. The percent inhibition was calculated, using the expression: (1 − A_i_/A_c_) × 100, where A_i_ and A_c_ are the absorbances obtained for AChE in the presence and absence of the inhibitors, respectively, after subtracting the respective background. Each experiment was performed in triplicate, and the mean ± standard deviation was calculated. Data from concentration-inhibition experiments of the inhibitors were calculated by nonlinear regression analysis, using the Excel program.

### 4.7. Water Permeability Measurements

Osmotic water permeability was measured in HeLa cells suspension by the stopped-flow light scattering method as previously described [22].

The antioxidant effect of compound **12** on water permeability HeLa cells were divided into different groups: (1) Controls, cells incubated at room temperature (21 °C); (2) heat-stressed cells, cells subjected to heat-treatment by incubating them in a water thermostatic and shacking bath at 42 °C for 3 h; (3) heat-stressed cells pre-treated, cells heat-stressed in the presence of the compound **12** at 20 µM final concentration (dissolved in methanol). Moreover, to test the possible capacity of the molecules to affect the AQP gating in normal condition, HeLa cells were treated in the presence and in the absence of the compounds by incubating at 21 °C for 3 h.

## Figures and Tables

**Table 1:** Glide Gscores, calculated Ligand Efficiencies and experimentally determined *K_i_* values of compounds **1**–**8** employed for the elaboration of the docking model.

Cmpd	State	Glide Gscore	Average Gscore	Ligand Efficiency (LE)	Average LE	*K_i_* S1R (nM) ± SEM ^a^	ln(*K_i_*)
**1**	diss	−10.560	−10.514	−0.4062	−0.4044	27 ± 1.8	3.296
	undiss	−10.467		−0.4026			
**2**	diss	−8.885	−8.885	−0.4039	−0.4039	15 ± 1.1	2.708
**3**	diss	−8.553	−8.553	−0.4277	−0.4277	2.2 ± 0.7	0.788
**4**	diss	−10.295	−9.188	−0.4680	−0.4176	2.9 ± 0.3	1.065
	undiss	−8.081		−0.3673			
**5**	diss	−9.101	−8.923	−0.4551	−0.4462	9 ± 0.7	2.197
	undiss	−8.745		−0.4373			
**6**	diss	−11.411	−10.548	−0.4075	−0.3767	340	5.829
	undiss	−9.685		−0.3459			
**7**	diss	−9.048	−9.048	−0.3619	−0.3619	478	6.170

^a^ Compounds with high affinity were tested three times. For compounds with low SR affinity (>250 nM), only one measure was performed.

**Table 2:** Relevant descriptors and properties predicted with QikProp.

Cmpd	MW	PSA	HBD	HBA	QPlogP_o/w_	QPlogS	QPlog HERG	QPP Caco	QPlog BB	Oral Abs ^b^
**8 ^a^**	279.38	30.874	0	4	3.51	−3.414	−6.564	1053.825	0.34	3
**9 ^a^**	309.40	38.211	0	4.75	3.924	−3.75	−7.077	1057.244	0.198	3
**9**	309.40	36.978	0	4.75	3.899	−3.604	−6.893	1049.048	0.202	3
**10**	323.43	38.951	0	4.75	4.112	−4.229	−7.246	972.122	0.085	3
**11**	281.39	31.865	0	4	3.612	−3.565	−6.442	984.507	0.242	3
**12**	307.43	32.094	0	4	4.268	−4.372	−6.98	971.725	0.156	3
**13**	337.46	39.309	0	4.75	4.28	−4.622	−7.356	899.612	−0.023	3
**Drug-likeness ^c^**	130–725	7.0–200	0–6	2–20	−2.0–6.5	−6.5–0.5	<−5	<25 poor >500 great	−3.0–1.2	1 low 2 med 3 high

^a^ Compounds computed as free bases. The other entries are protonated species. ^b^ Predicted qualitative human oral absorption: 1, 2, or 3 for low, medium, or high. The assessment uses a knowledge-based set of rules, including checking for suitable values of PercentHumanOralAbsorption, number of metabolites, number of rotatable bonds, logP, solubility and cell permeability. ^c^ Range or recommended values of each property for comparison with those of 95% of known drugs.

**Table 3:** LE, predicted and experimental binding affinities of compounds **8**–**13** toward S1R binding site.

cmpd	LE	Predicted ln(*K_i_*)	Predicted *K_i_* (nM)	Experimental *K_i_* S1R (nM) ± SEM	Experimental ln(*K_i_*)
**8**	−0.39271	3.9754	44.92	132 ± 55	4.8828
**9**	−0.40309	3.3053	21.10	48 ± 12	3.8712
**10**	−0.40696	3.0555	15.92	25 ± 7	3.2189
**11**	−0.45271	0.1022	0.57	4.4 ± 2.4	1.4816
**12**	−0.43748	1.0854	1.72	33 ± 14	3.4965
**13**	−0.36672	5.6531	298.28	87 ± 28	4.4659

## Abbreviations

AChE	Acetylcholinesterase
ADME	Absorption, distribution, metabolism and excretion
AQP	Aquaporin
BBB	Blood–brain barrier
CNS	Central nervous system
ER	Endoplasmic reticulum
IFD	Induced fit docking
LE	Ligand efficiency
MAM	Mitochondria-associated ER membrane
NMDA	*N*-methyl-d-aspartate
NMDAR	*N*-methyl-d-aspartate receptor
RMSD	Root-mean-square deviation
ROS	Reactive oxygen species
S1R	Sigma-1 receptor
S2R	Sigma-2 receptor
SEM	Standard error of the mean
